# Eubacterial SpoVG Homologs Constitute a New Family of Site-Specific DNA-Binding Proteins

**DOI:** 10.1371/journal.pone.0066683

**Published:** 2013-06-20

**Authors:** Brandon L. Jutras, Alicia M. Chenail, Christi L. Rowland, Dustin Carroll, M. Clarke Miller, Tomasz Bykowski, Brian Stevenson

**Affiliations:** 1 Department of Microbiology, Immunology, and Molecular Genetics, University of Kentucky College of Medicine, Lexington, Kentucky, United States of America; 2 Department of Agricultural Sciences, University of Kentucky College of Agriculture, Lexington, Kentucky, United States of America; 3 Graduate Center for Toxicology, University of Kentucky College of Medicine, Lexington, Kentucky, United States of America; 4 Brown Cancer Center, University of Louisville, Louisville, Kentucky, United States of America; University of Toledo School of Medicine, United States of America

## Abstract

A site-specific DNA-binding protein was purified from *Borrelia burgdorferi* cytoplasmic extracts, and determined to be a member of the highly conserved SpoVG family. This is the first time a function has been attributed to any of these ubiquitous bacterial proteins. Further investigations into SpoVG orthologues indicated that the *Staphylococcus aureus* protein also binds DNA, but interacts preferentially with a distinct nucleic acid sequence. Site-directed mutagenesis and domain swapping between the *S. aureus* and *B. burgdorferi* proteins identified that a 6-residue stretch of the SpoVG α-helix contributes to DNA sequence specificity. Two additional, highly conserved amino acid residues on an adjacent β-sheet are essential for DNA-binding, apparently by contacts with the DNA phosphate backbone. Results of these studies thus identified a novel family of bacterial DNA-binding proteins, developed a model of SpoVG-DNA interactions, and provide direction for future functional studies on these wide-spread proteins.

## Introduction

To be successful, single-celled organisms must efficiently and rapidly adapt to changing conditions. This is often accomplished through exquisite regulatory networks involving numerous, dynamic trans-acting factors. Prokaryotic proteins that bind to nucleic acids govern virtually every cellular process, including nucleoid organization, transcription, translation, and DNA replication, modification, repair, and recombination. Remarkably, most DNA-binding proteins are poorly characterized, and it is likely that many more await discovery.

In our studies of the VlsE antigenic variation system of *Borrelia burgdorferi*, the causative agent of Lyme disease [1], [2], we discovered that these bacteria produce a cytoplasmic protein which specifically binds a DNA site within the *vlsE* open reading frame. Using a powerful, unbiased approach, we identified that protein to be the borrelial SpoVG. A broad range of Eubacteria, including many important human pathogens, encodes homologs of SpoVG. The name derives from observations that *Bacillus* spp. *spoVG* mutants are unable to complete stage five of sporulation [3]–[5]. *Bacillus* spp. mutants exhibit additional defects, such as abnormal cell cycle and division [4], [6], [7]. *Staphylococcus aureus spoVG* mutants are less virulent than are wild-type bacteria, and produce significantly lower levels of several pathogenesis-related factors [8]–[10]. With many organisms, production of *spoVG* is developmentally regulated and often utilizes alternative sigma factors [11]–[23]. The three dimensional structures have been determined for SpoVG from *S. aureus* and other species, and found to be very highly conserved ([24], and Protein Data Base [PDB] accession numbers 2IA9, 2IA9X, 2IA9Z). However, until our discovery, the biochemistry of SpoVG remained a mystery.

Here we demonstrate that the SpoVG homologues of *Borrelia burgdorferi, Staphylococcus aureus,* and *Listeria monocytogenes* all bind to DNA. Further investigations determined that, while SpoVG members are highly similar, they have evolved to bind different consensus sequences. Alanine mutagenesis and domain shuffling revealed residues and microdomains required for generalized DNA binding and for nucleotide sequence specificity.

## Results

### Identification of *B. burgdorferi* SpoVG as a Site-specific DNA-binding Protein

**Figure 1:**
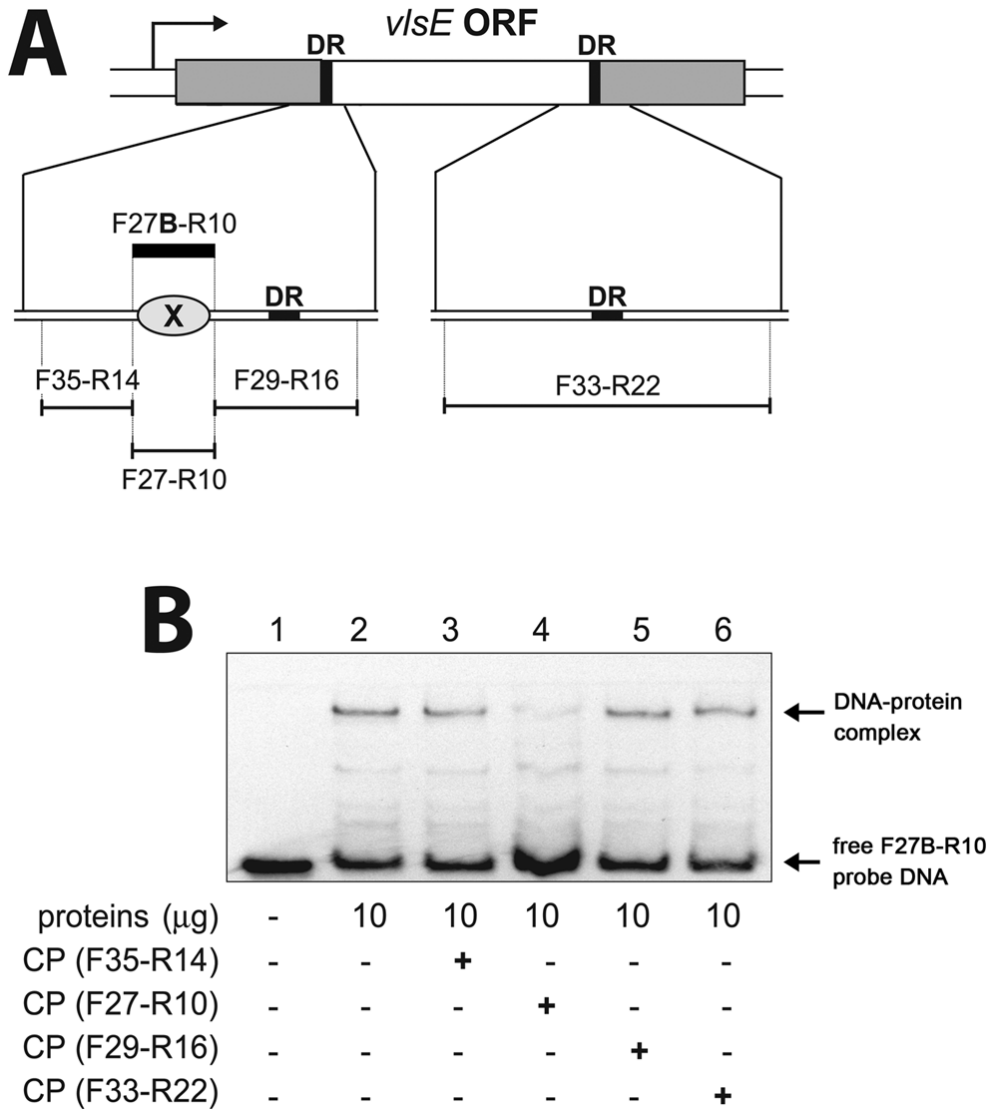
A *B. burgdorferi* cytoplasmic protein binds DNA within the *vlsE* open reading frame. (**A**) Schematic of the *vlsE* expression locus and DNAs uses for EMSA. In the upper illustration of the *vlsE* ORF, the gray areas represent the invariable regions, the white area represents the hypervariable region, and the black bars indicate the direct repeat sequences. Below that, the location of each labeled or unlabeled EMSA DNA is represented by thick or thin black horizontal lines, respectively. “DR” indicates the directly-repeated sequences bordering the variable region of *vlsE*. (**B**) EMSAs using *B. burgdorferi* cytoplasmic extracts. Lane 1–6∶1 nM of labeled probe F27B-R10. Lane 2–6∶10 µg cell-free cytoplasmic extract. Lane 3∶100-fold molar excess unlabeled competitor F35-R14. Lane 4∶100-fold molar excess unlabeled competitor F27–R10. Lane 5∶100-fold molar excess unlabeled competitor F29–R16. Lane 6∶100-fold molar excess unlabeled competitor F33–R22.

As part of our studies of the *vlsE* system, we postulated that *B. burgdorferi* expresses a cytoplasmic factor(s) that binds near the recombination site, to help control genetic rearrangement. Addressing that hypothesis, we observed that incubating cell-free *B. burgdorferi* cytoplasmic extract with *vlsE* DNA retarded the electrophoretic mobility of DNA, consistent with a DNA-protein complex (Fig. 1). This complex was not evident when cytoplasmic extracts were heat denatured or treated with proteinase K, indicating the need for a properly folded, intact protein (data not shown). Additional EMSAs narrowed the protein-binding site even further. The 70 bp labeled probe F27B-R10 bound the cytoplasmic protein, and binding was competed by the unlabeled version of that DNA sequence, fragment F27–R10 (Fig. 1A and B, lane 4). DNAs flanking those 70 bp did not compete for protein binding (Fig. 1B, lanes 3 and 5). These results indicate that the borrelial protein binds a DNA sequence of approximately 70 bp (X on Fig. 1A), and that neither of the repeat regions flanking the recombination site is involved in protein binding.

**Figure 2:**
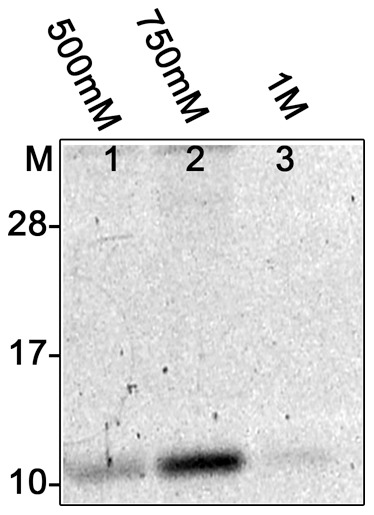
DNA-affinity chromatographically purified SpoVG*_Bb_*. *vlsE* probe affixed to magnetic beads was incubated with cell-free extracts and bound protein were eluted by titration with increasing concentrations of NaCl. Eluates were separated by SDS PAGE, stained with Sypro Ruby. M. Molecular mass standards. Lanes 1–3. Proteins eluted with 500, 750, and 1000 mM NaCl elution, respectively.

To identify the unknown factor, we took advantage of a DNA affinity chromatography method developed in our laboratory, which has identified several other novel sequence-specific DNA-binding proteins [25]–[27]. Using a segment of *vlsE* that included the high-affinity binding site as bait, a protein of approximately 12 kDa was purified. Buffers containing at least 500 mM NaCl were required to elute the protein off the DNA, indicating that the trans acting factor had a high affinity for *vlsE* bait DNA (Fig. 2). Matrix assisted laser deionizing- time of flight (MALDI-TOF) MS/MS analysis identified the protein as being encoded by open reading frame BB0785, a hypothetical protein of unknown function, with a corresponding Mascot score of 212. Control reactions that used the same cell-free extracts and different DNA baits did not pull-down this protein (data not shown).

**Figure 3:**
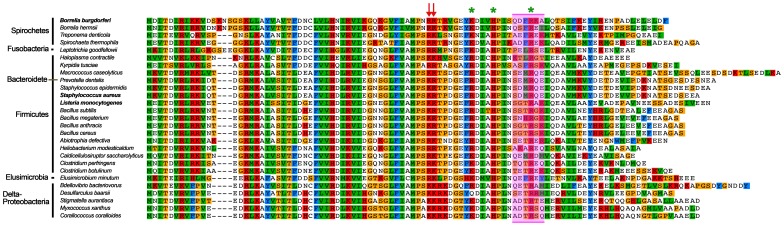
SpoVG is a highly conserved Eubacteria protein. **Illustrated is an alignment of the predicted sequences of SpoVG proteins from 19 different families of 6 different phyla.** Alignment was performed using Geneious software, Pfam 200, with 1000 iterations. Identical or homologous amino acids found in the same position of multiple proteins are indicated by same-colored boxes. Note that these analyses grouped the SpoVG protein of the opportunistic oral pathogen *Prevotella dentalis* with the Gram-positive *Bacilli* class, although it is currently considered to be a member of the *Bacteroides*. Consistent with these results, *P. dentalis* has morphological and biochemical features which differ from other species in the genus *Prevotella* and class *Bacteroides* [51]. Red arrows indicate residues demonstrated to be involved in SpoVG-DNA interactions. Green asterisks denote conserved residues that were found to be not required for binding DNA. The magenta box indicates residues of SpoVG*_Bb_* and SpoVG*_Sa_* involved in DNA sequence specificity.

**Figure 4:**
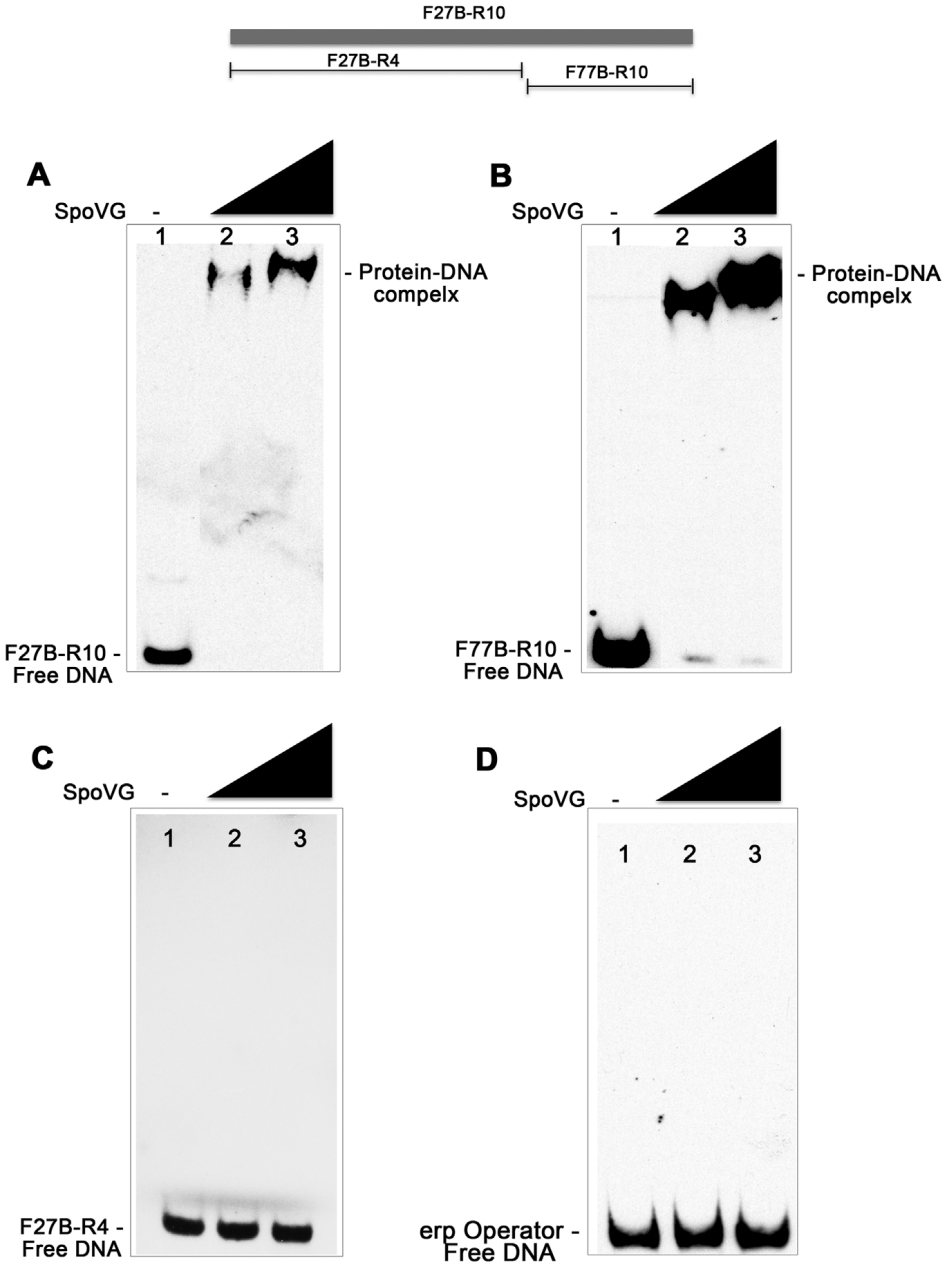
Identification of a SpoVG***_Bb_*** high-affinity binding site. Recombinant SpoVG*_Bb_* binds specifically to an 18 bp sequence of the *vlsE* open reading frame. (**A**) Lanes 1–3∶1 nM of labeled F27B-R10. Lane 2∶300 nM SpoVG*_Bb_*. Lane 3∶600 nM SpoVG*_Bb_*. (**B**) Lanes 1–3∶1 nM of labeled F77B-R10. Lane 2∶300 nM SpoVG*_Bb_*. Lane 3∶600 nM SpoVG*_Bb_*. (**C**) Lanes 1–3∶1 nM of labeled F27B-R4. Lane 2∶300 nM SpoVG*_Bb_*. Lane 3∶600 nM SpoVG*_Bb_*. (**D**) Lanes 1–3∶1 nM of labeled *erp* Operator DNA. Lane 2∶300 nM SpoVG*_Bb_*. Lane 3∶600 nM SpoVG*_Bb_*.

Due to its homology with the SpoVG proteins of many bacterial species, we have retained that name for the borrelial gene and protein (Fig. 3). To confirm that this protein was responsible for the protein-DNA complex formed by cytoplasmic extracts, we purified recombinant *B. burgdorferi* SpoVG (SpoVG*_Bb_*) and repeated the EMSAs. Indeed, recombinant SpoVG*_Bb_* bound to probe F27B-R10 (Fig. 4A). This 70-mer was dissected and one 18 bp fragment was found to be required and sufficient for SpoVG-binding (Figs. 4B & C). SpoVG*_Bb_* bound to its high-affinity target DNA with an apparent disassociation constant (K_D_) of 308 (±31) nM. Further controls incorporated *erp* Operator DNA, a region of DNA known to be bound by other *B. burgdorferi* DNA-binding proteins [25], [28], [29]; SpoVG*_Bb_* failed to bind this sequence, confirming its specificity for *vlsE* DNA (Fig. 4D). The identified SpoVG*_Bb_*-binding sequence does not occur anywhere else in the *B. burgdorferi* genome, although it is possible that this protein may bind sequences that differ slightly from the site within *vlsE*. These studies were the first to demonstrate a function for a SpoVG orthologue. The role(s) of SpoVG*_Bb_* in *vlsE* rearrangement is still under investigation, and is beyond the scope of this communication on the biochemistry of SpoVG-DNA interactions.

### *S. aureus* SpoVG is also a Site-specific DNA-binding Protein

**Figure 5:**
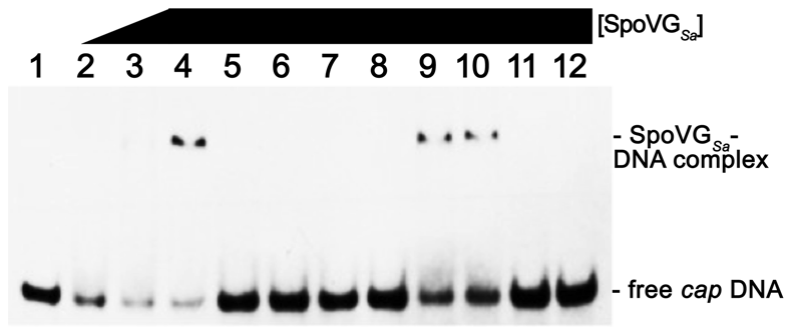
SpoVG*_Sa_* binds specifically to *S. aureus cap5, fmtB, lukED,* and *saeX* DNAs adjacent to the promoter. Illustrated are EMSAs with *S. aureus* SpoVG*_Sa_*, labeled *cap5* 5′ non-coding DNA, and various unlabeled competitor DNAs. **Lanes 1–12**∶5 nM of labeled *capA* promoter DNA. **Lanes 2–4**: Increasing amounts of SpoVG*_Sa_* (0.2 µg, 0.4 µg, and 0.6 µg, respectively). **Lanes 5–12**∶0.8 µg of SpoVG*_Sa_*. **Lanes 5–8**∶50-fold molar excess of unlabeled *fmtB, lukED, esxA,* or *cap5* 5′ non-coding DNAs, respectively. **Lane 9**∶50-fold molar excess of *esxA* ORF DNA. **Lane 10**∶50-fold molar excess of *cap5A* ORF DNA. **Lane** **11:** SpoVG*_Sa_* was heated to 99°C for 5 minutes before use in EMSA. **Lane 12**: SpoVG*_Sa_* was preincubated with 5 mg/ml of Proteinase K before use in EMSA.

Bioinformatics indicate that many spore and non-spore forming bacteria, Gram positive and Gram negative, encode a SpoVG protein (Fig. 3). Given the high degree of sequence conservation, we hypothesized that these orthologues also bind DNA. Compared to wild-type bacteria, *S. aureus* s*poVG* mutants express significantly less *capA-H* mRNA and synthesize reduced levels of capsule [9], [17]. We hypothesized that *S. aureus* SpoVG (SpoVG*_Sa_*) might bind DNA near the *cap* operon promoter. This was confirmed by EMSA, which demonstrated that recombinant SpoVG*_Sa_* bound to *S. aureus* (Newman) *cap5* 5′-non-coding DNA in a dose dependent fashion (Fig. 5, lanes 2–4). Heat denaturation or proteinase K treatment eliminated the shifted EMSA band, confirming that this complex contained functional protein (Fig. 5, lanes 11 and 12). In order to determine the relative affinity of the SpoVG*_Sa_*-DNA interaction, three independent protein preparations and multiple EMSAs with labeled *cap* probe were performed with saturating concentrations of SpoVG*_Sa_*. These experiments indicated an average K_D_ of 316 (±42) nM.

In a whole transcriptome screen of a *S. aureus spoVG* mutant, significant alterations in several other virulence-related loci were documented, including *fmtB, esxA,* and *lukED* [9]. The ability of SpoVG*_Sa_* to bind near the promoters of those genes was evaluated using each DNA as an unlabeled EMSA competitor against labeled *cap5* DNA. The *fmtB, esxA,* and *lukED* 5′ non-coding DNAs each competed with labeled *cap5* probe for binding of SpoVG*_Sa_* (Fig. 5, lanes 5–7). Control studies using unlabeled competitors derived from the *esxA* or *cap5A* open reading frames had substantially lesser effects on SpoVG*_Sa_* binding to the labeled *cap5* probe (Fig. 5, lanes 9 and 10). These results indicate that the 5′-non-coding regions of *cap5, fmtB, esxA,* and *lukED* all contain a unique sequence(s) to which SpoVG*_Sa_* binds with high affinity and specificity.

**Figure 6:**
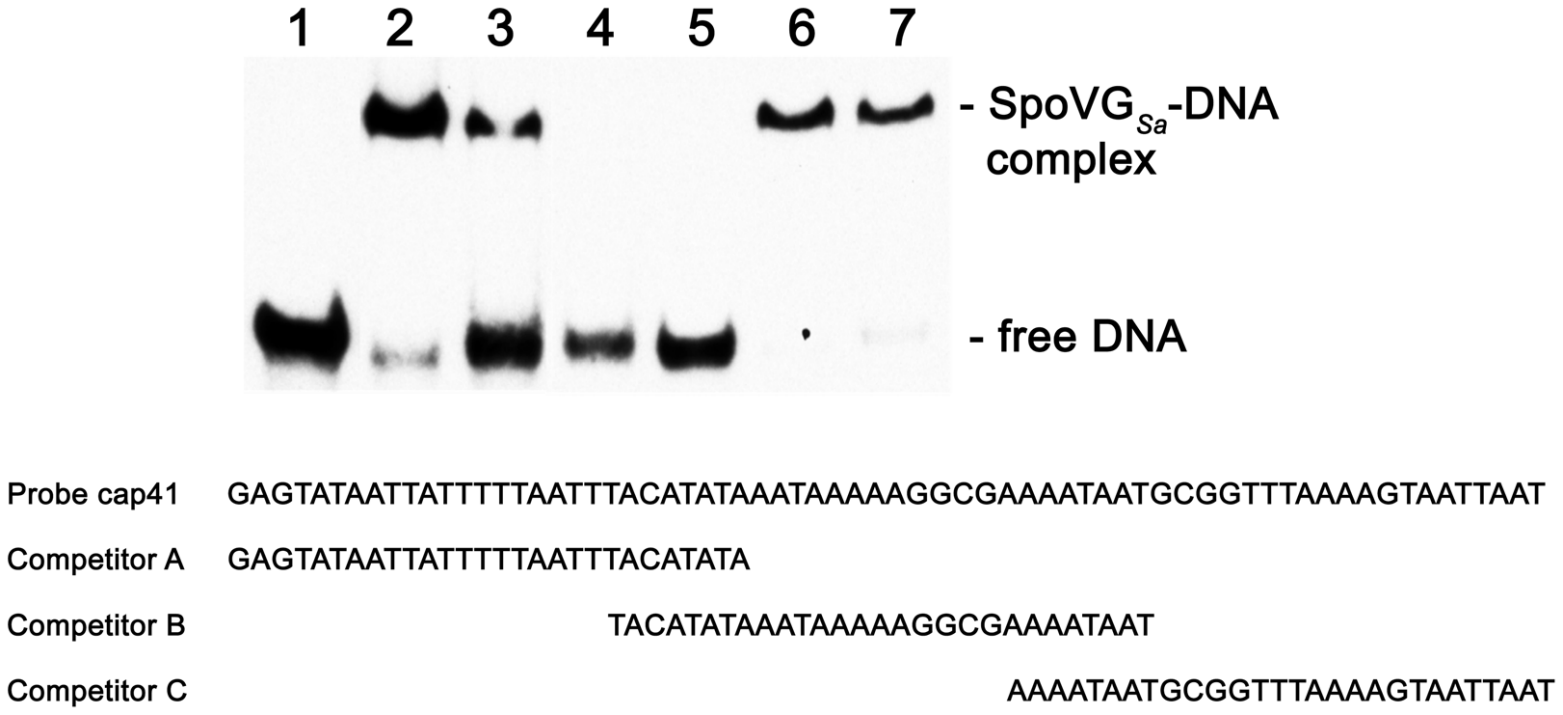
SpoVG*_Sa_* interacts specifically with a 28 bp region adjacent to the *cap5* promoter. EMSA with a labeled 62 bp probe derived from of *cap5* 5-non-coding DNA (cap41) and different concentration of 28 bp cold competitors. **Lanes 1–7**∶7.5 nM cap41 probe (−142 through −84 from translational start site). **Lanes 2–7∶**0.8 µg SpoVG. **Lane 3–5∶**10-, 25-, and 50-fold molar excess of competitor A respectively. **Lane 6**∶50-fold molar excess of competitor B. **Lane 7**∶50-fold molar excess of competitor C.

Additional EMSAs using a smaller probe and unlabeled competitors narrowed down the high-affinity SpoVG*_Sa_*-binding sequence in *cap5* promoter-proximal DNA. Probe cap41 contains a SpoVG-binding site (Fig. 6, lane 2). Three unlabeled 28 bp DNAs, which span the 62 bp sequence of probe cap41, were included in EMSAs at molar excesses over probe cap41. This type of analysis prevents a possible bias towards probe and/or competitor length, while controlling for potential high affinity interactions at the ends of the probe. At a constant concentration of SpoVG*_Sa_*, addition of competitor A decreased the amount of bound probe and increased the amount of free DNA (Fig. 6, lanes 3–5). In contrast, 5-fold greater concentrations of competitors B or C did not detectably affect SpoVG*_Sa_* binding to probe cap41 (Fig. 6, lanes 6 and 7). These data indicate that the high affinity-binding site is contained within the 28 nucleotides of competitor A.

**Figure 7:**
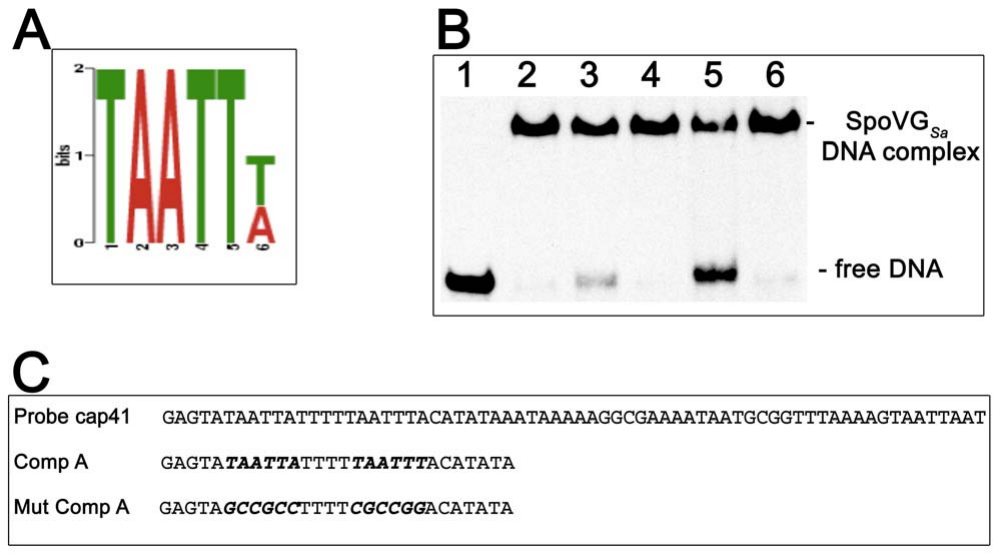
The palindromic motif 5′-ATTAA^T^/_A_-3′ is required for SpoVG***_Sa_*** binding. (**A**) The conserved sequence, 5′ to 3′, identified by multiple motif analysis of promoters bound to and influenced by SpoVG*_Sa_*. (**B**) EMSA with a labeled probe cap41, derived from *cap5* sequence, and two different 28 bp cold competitors. All lanes contain 5 nM labeled cap41 DNA. Lanes 2–6 also include 0.9 µg of SpoVG*_Sa_*. Lanes 3 and 4∶25 fold molar excess competitor A or mutant competitor A, respectively. Lanes 5 and 6∶50 fold molar excess of competitor A and mutant competitor A, respectively. (**C**) Sequences of probe *cap41* and competitors. The differences between the wild type and mutant competitors are indicated by boldface italics.

MEME (Multiple Em for Motif Elicitation) analyses of the DNAs bound by SpoVG*_Sa_* indicated that all contain at least two 5-TAATT^T^/_A_-3′ sequences (Fig. 7A). Competitor A contains two copies of that motif. To evaluate whether this motif is involved with SpoVG binding, a competitor with mutated motifs was incorporated into subsequent EMSAs (Fig. 7C). SpoVG*_Sa_* exhibited greater than five-fold higher affinity for the wild-type competitor over the mutant (Fig 7B). Taken together, these results demonstrate that the *S. aureus* SpoVG protein preferentially binds to DNA containing an TAATT^T^/_A_ motif. Whether SpoVG*_Sa_* will bind to any such sequence or if surrounding DNA sequences/structures contribute to protein binding remains to be determined.

### Different SpoVG Homologues Bind to Different DNA Sequences

**Figure 8:**
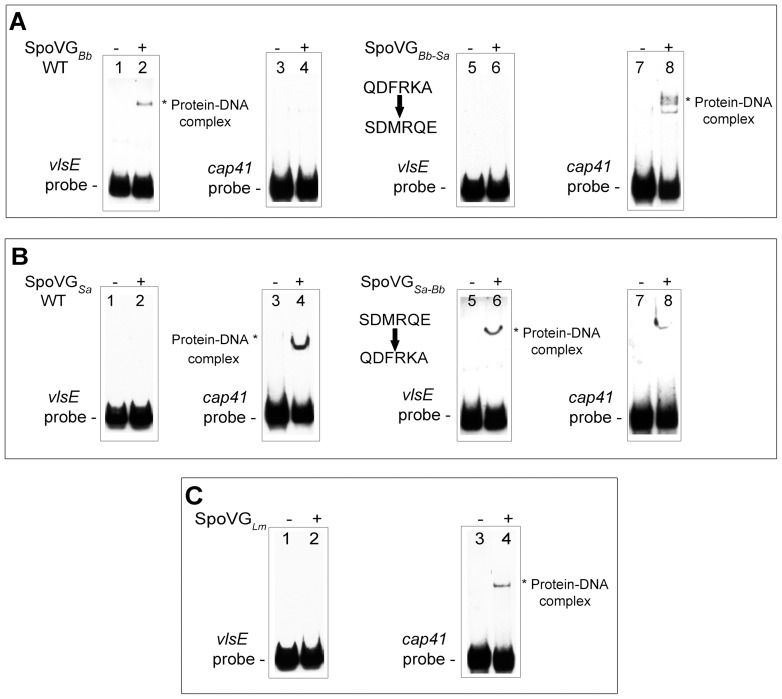
Domain swapping experiments defined a region of SpoVG required for nucleotide-binding specificity. *B. burgdorferi* SpoVG*_Bb_* residues QDFRKA were mutated to the analogous residues of *S. aureus* to produce SpoVG*_Bb-Sa_*. Similarly, SpoVG*_Sa_* residues SDMRQE were changed to the corresponding *B. burgdorferi* SpoVG*_Bb_* residues to create SpoVG*_Sa-Bb._* These chimeric proteins were queried for their respective abilities to bind *vlsE* and *cap5* probes. (**A**) SpoVG*_Bb-Sa_* domain swap. Lanes 1, 2, 5, and 6 contain labeled *vlsE* probe. Lanes 3, 4, 7, and 8 contain labeled *cap5* probe. Lane 2 and 4, 200 nM wild-type SpoVG*_Bb_.* Lane 4 and 8, 200 nM SpoVG*_Bb-Sa_*. (**B**) SpoVG*_Sa-Bb_* domain swap. Lanes 1, 2, 5, and 6 contain 5 nM of labeled *vlsE* probe. Lanes 3, 4, 7, and 8 contain labeled *cap5* DNA. Lane 2 and 4, 200 nM wild-type SpoVG*_Sa_.* Lane 4 and 8, 200 nM SpoVG*_Sa-Bb_*. (**C**) *Listeria monocytogenes* SpoVG binds *cap5* DNA. Lanes 1 and 2 include 5 nM *vlsE* probe DNA, Lanes 3 and 4 contain 5 nM *cap5* probe DNA, and Lanes 2 and 4 contain 200 nM SpoVG*_Lm_* protein.

The *vlsE* probe, to which SpoVG*_Bb_* binds with high-affinity and specificity, does not possess the SpoVG*_Sa_* consensus binding motif. These observations suggested that SpoVG homologues might bind to divergent, distinct DNA sequences. With this in mind, we incubated equal concentrations of SpoVG*_Sa_* or SpoVG*_Bb_* with labeled *vlsE* and *cap41* probes in independent EMSAs. SpoVG*_Bb_* bound to the *vlsE* probe, but not *cap41* (Fig. 8A). Likewise, SpoVG*_Sa_* bound to only the *cap41* probe (Fig. 8B).

To further address our hypothesis that SpoVG homologues act in a similar fashion, but interact with different sequences, we purified the SpoVG homologue from another firmicute, *Listeria monocyotgenes* (SpoVG*_Lm_*). SpoVG*_Lm_* bound *S. aureus cap41* promoter DNA but not *B. burgdorferi vlsE* DNA (Fig. 8C).

### Chimeric SpoVG Proteins Identify Residues Involved with Sequence Specificity

Orthologous proteins are under selective pressure to maintain function, but can diverge in amino acid composition to accommodate the needs of the individual species. Protein structural predictions indicated that SpoVG homologues possess a hypervariable alpha helix at the carboxy terminus (Fig. 3). We suspected that it was this variable domain that contributed to the above-described DNA sequence specificity. To address this hypothesis, we created two different chimeric SpoVG proteins. The staphylococcal SpoVG protein was mutated at residues S^66^ through E^71^ and changed to the corresponding borrelial SpoVG residues, creating the chimeric variant SpoVG*_Sa-Bb_*. We reciprocated this strategy by exchanging residues Q^69^ through A^74^ of SpoVG*_Bb_* with those of the *S. aureus* protein, generating the chimeric protein SpoVG*_Bb-Sa_* (Fig 8AB and Fig. 3). For both chimeras, exchanging 6 residues was sufficient to permit binding to the alternative consensus sequence. SpoVG*_Bb-Sa_* bound to the cap41 probe, but could no longer bind to the *vlsE* probe. The SpoVG*_Sa-Bb_* protein now bound *vlsE* DNA. That chimera retained a slight ability to interact with the cap41 DNA, albeit at a dramatically reduced affinity for which a K_D_ could not be calculated (Fig. 8B). Taken together, these results demonstrate that sequence divergence within the alpha helix contributes to DNA sequence specificity.

### Conserved Residues Essential for DNA-protein Complexes

Bacterial proteins that perform analogous functions often retain similar biochemical and structural features in order to interact with their respective ligands [30]. We reasoned that, since three different SpoVG proteins interact with DNA, conserved residues common to all SpoVG orthologues might be required for non-specific substrate binding. Recombinant SpoVG*_Sa_* and SpoVG*_Bb_* proteins were produced that included single or double amino acid substitutions at conserved positions (Fig. 3. and Table 1). These mutant proteins were tested for their abilities to interact with their respective high-affinity DNA sequences.

**Table 1:** Plasmids used in this study.

Plasmid Name	Backbone	Organism/Target locus	Description
pBLJ340	pET101	*L.monocytogenes/spoVG*	rSpoVG
pBLJ117	pET101	*B.burgdorferi/spoVG*	rSpoVG
pBLJ341	pET101	*B.burgdorferi/*Δ*spoVG*	ΔSpoVG R53A, R54A
pBLJ342	pET101	*B.burgdorferi/*Δ*spoVG*	ΔSpoVG H65A
pBLJ343	pET101	*B.burgdorferi/*Δ*spoVG*	ΔSpoVG R72A
pBLJ347	pET101	*B.burgdorferi/*Δ*spoVG*	ΔSpoVG BbQDFRKA Sa SDMRQE
pBLJ505	pET101	*S. aureus/spoVG*	
pBLJ351	pET101	*S. aureus/*Δ*spoVG*	ΔSpoVG K50A, R51A
pBLJ352	pET101	*S. aureus/*Δ*spoVG*	ΔSpoVG H62A
pBLJ353	pET101	*S. aureus/*Δ*spoVG*	ΔSpoVG R69A
pBLJ354	pET101	*S. aureus/*Δ*spoVG*	ΔSpoVG K50A
pBLJ355	pET101	*S. aureus/*Δ*spoVG*	ΔSpoVG R51A
pBLJ357	pET101	*S. aureus/*Δ*spoVG*	ΔSpoVG SaSDMRQE BbQDFRKA
pBLJ506	pCR2.1	*S. aureus/cap5A*	*cap5′* UTR
pBLJ507	pCR2.1	*S. aureus/fmt*	*fmtB* UTR
pBLJ508	pCR2.1	*S. aureus/esxA*	*esxA* UTR
pBLJ509	pCR2.1	*S. aureus/luk*	*lukED* UTR
pTB7a	pCR2.1	*B. burgdorferi/vlsE*	*vlsE* −80 +240

**Figure 9:**
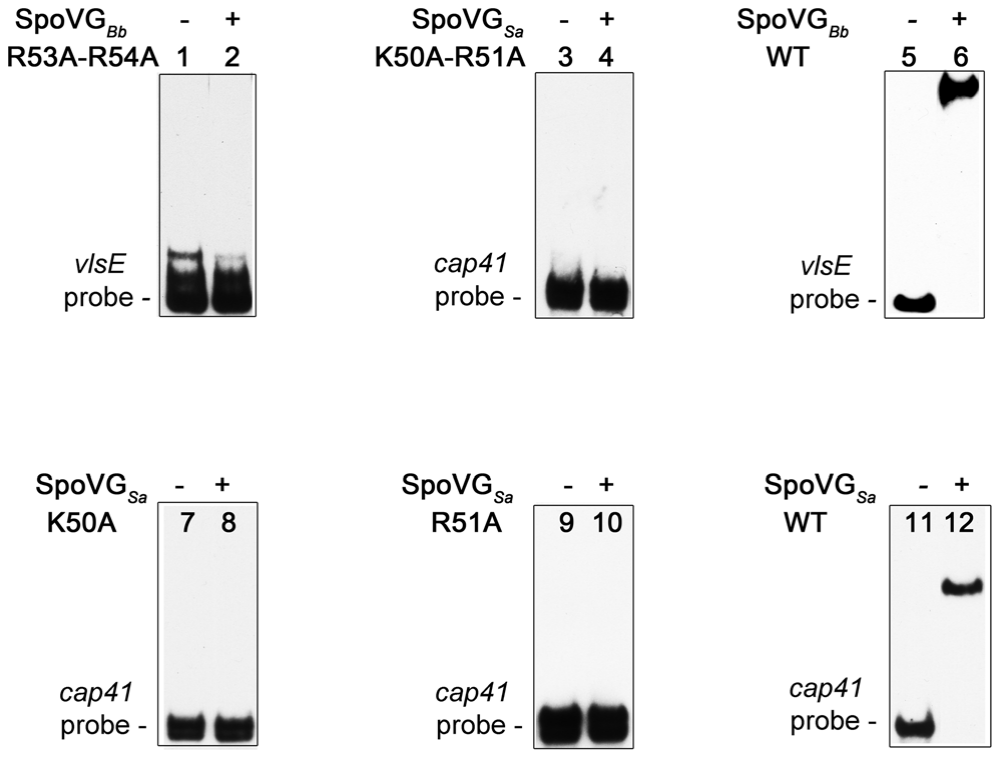
Alanine mutagenesis determined residues required for binding DNA. Lanes 1, 2, 5 and 6 contain 2 nM labeled *vlsE* probe. Lanes 3,4, and 7–12 contain 2 nM labeled *cap41* probe. Additional ingredients of each EMSA are: Lane 2, 1.5 µM mutant SpoVG*_Bb_* R53A-R54A; Lane 4, 1.5 µM mutant SpoVG*_Sa_* K50A-R51A; Lane 6, 500 nM wild-type SpoVG*_Bb_*; Lane 8, 1.5 µM mutant SpoVG*_Sa_* K50A: Lane 10, 1.5 µM mutant SpoVG*_Sa_* R51A; Lane 12, 500 µM wild-type SpoVG*_Sa_*.

Initial investigations targeted a doublet of positively charged residues (R and K), which were conserved in all SpoVG homologues (Fig. 3). The two charged residues are predicted to project inward from an abbreviated β-sheet, toward the carboxy-terminal alpha helix. Alanine substitutions at position R53–R54 of SpoVG*_Bb_* or K50–R51 of SpoVG*_Sa_* impaired DNA binding. Addition of mutant proteins at five-fold excess over the disassociation constant of the wild-type protein still did not produce a detectable EMSA shift (Figs. 3 and 9). To assay residues independently, SpoVG*_Sa_* K50A and SpoVG*_Sa_* R51A were created. These variants exhibited the same deficiency in DNA binding as did the double mutant, confirming that both conserved residues are required for DNA binding.

Mutations to other conserved, positively charged residues did not have any significant effects on DNA binding (Fig. 3, Table 1, and data not shown). Additionally, none of the other mutant proteins exhibited altered sequence preference (data not shown).

### Site-directed Mutagenesis did not Affect Multimerization

**Figure 10:**
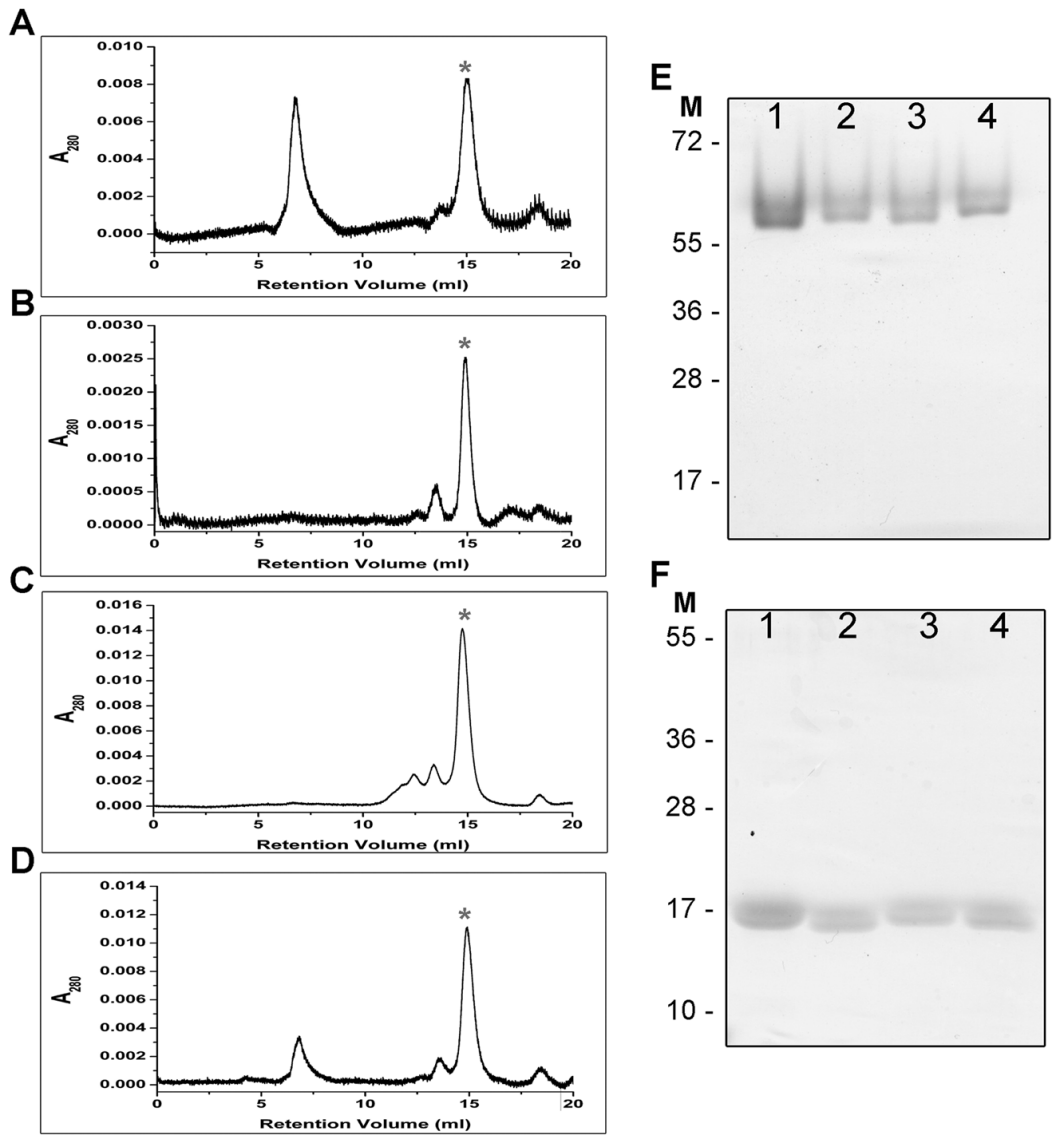
Site directed mutagenesis did not influence SpoVG oligomerization. Results of panels A through D illustrate HPLC sizing column chromatography of wild type and mutant SpoVG*_Sa_* proteins. For some preparations, proteins eluted with a retention time of approximately 7 minutes, which corresponds with a molecular mass >440 kDa and are composed of protein aggregates. (**A**) Wild-type SpoVG*_Sa_*; (**B**) SpoVG*_Sa_* K50A-R51A; (**C**) SpoVG*_Sa_* K50A; (**D**) SpoVG*_Sa_* R51A. Peaks marked with red asterisks indicate retention volumes corresponding with approximately 55–60 kDa. Panels E and F illustrates proteins separated following native of denaturing PAGE, respectively. M, Molecular mass standards; Lane 1, 1.5 µM mutant SpoVG*_Sa_* K50A-R51A; Lane 2, 1 µM mutant SpoVG*_Sa_* K50A; Lane 3, 1 µM mutant SpoVG*_Sa_* R51A; Lane 4, 1 µM wild-type SpoVG*_Sa_*.

Replacing charged or polar residues with a small, non-polar, uncharged alanine can interfere with protein-protein interactions, or cause protein misfolding [31]. To that end, sizing chromatography and tandem native/denaturing PAGE analysis were used to examine the native state of SpoVG*_Sa_*. The recombinant protein has a molecular mass of 14.6 kDa. By two independent methods, our data indicate that SpoVG*_Sa_* forms a 55–60 k Da complex in solution, consistent with a tetramer (Fig. 10). The complexes disappeared when samples were denatured, demonstrating that these bands were not the results of contamination (Fig. 10C). None of the SpoVG*_Sa_* mutants exhibited diminished multimer formation, suggesting that the mutants which were impaired for DNA binding still retained their ability to fold correctly and form higher ordered species in solution.

## Discussion

The current studies yielded several novel findings that impact a broad range of Eubacterial species. First, SpoVG orthologues from three distinct bacteria bound DNA. For several bacterial species, it is known that these small proteins play key roles in critical cellular processes, which we can now hypothesize are due to SpoVG-DNA interactions. Second, these discoveries help explain why SpoVG was found in association with the *S. aureus* nucleoid, and the involvement of the *Bacillus subtilis* orthologue with nucleoid organization [4], [32]. Third, while SpoVG proteins are highly conserved overall, the *S. aureus* and *B. burgdorferi* proteins interact preferentially with distinct DNA sequences. Given the amino acid divergence among different orthologs’ carboxy-terminal alpha helices, we speculate that this feature may also be true for other SpoVG homologues. Finally, we identified two residues, whose biochemical properties are conserved among SpoVGs, that are essential for DNA interactions.

**Figure 11:**
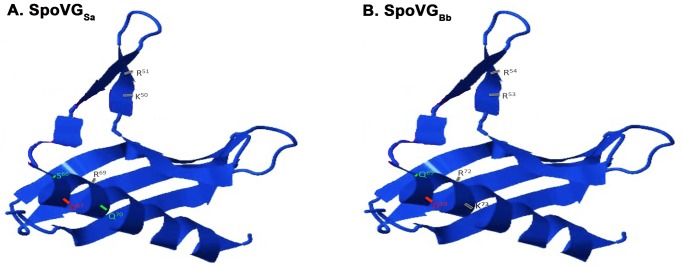
Monomeric structures of SpoVG*_Sa_* and SpoVG*_Bb,_* modeled on the solved *S. epidermidis* SpoVG protein structure. Residues required for DNA-binding and those involved with sequence specificity are indicated, with different colors reflecting biochemical properties of amino acids: Gray = positively charged, Red = negatively charged, and Green = polar, uncharged. (**A**) SpoVG*_Sa_*. (**B**) SpoVG*_Bb_*.

Residues involved with maintaining SpoVG secondary structure model well between species, suggesting that the solved crystal structures are likely to be representative of all orthologous proteins. Merging all of these data, we propose a model for SpoVG binding (Fig. 11). Solvent-accessible, positively charged residues are located adjacent to the alpha helix and can stabilize duplex binding through electrostatic interactions with the phosphate backbone of DNA. These are residues R53 and R54 of SpoVG*_Bb_*, and K50 and R51 of SpoVG*_Sa_*. Those residues extend into a pocket, while the alpha helix is arranged perpendicularly to provide base-edge specificity through interactions by residues extending into the pocket (Figs. 10 and 11). Notably, the *B. burgdorferi* and *S. aureus* alpha helices are out of phase by approximately one turn of the alpha helix, presenting residues with dissimilar hydrogen-donating and hydrogen-accepting capabilities on the upper helical face ([31], and Fig. 11). Independent evolution of the two studied SpoVG proteins resulted in different nucleic acid binding specificity. Our data suggest that SpoVG homologs of different bacterial species may bind to distinct DNA sequences, and possibly exert different effects on physiology. Similar phenomena have been documented that alter the specificity, diversify the signal, and eliminate unwanted cross-talk between sensor histidine kinases and response regulators in two-component signal transduction systems [33]–[35].

The mechanisms by which *S. aureus* controls of virulence-associated genes are poorly understood. The identification of a SpoVG*_Sa_*-binding site adjacent to the *cap* promoter suggests that SpoVG*_Sa_* may play a direct role in controlling capsule production. Indeed, *cap* transcription is significantly reduced in *spoVG* mutant *S. aureus* [9], and *S. aureus* lacking a SpoVG*_Sa_*-binding site in the *cap* promoter exhibit reduced *cap* transcription [36]. However, expression of the *cap* operon has been reported to be controlled by at least 12 other regulatory factors [9], [17], [36]–[40]. Studies are currently under way to define binding-sites of the other regulatory factors, and to understand the ways in which these many regulators interact with each other and with RNA polymerase to control *cap* expression.

The role of SpoVG*_Bb_* in *B. burgdorferi vlsE* genetic rearrangement remains to be determined. The specialized recombination processes involved are complex and highly regulated, occurring only during mammalian infection but never during tick colonization or in culture [2], [41]. Recombination of *vlsE* is RecA-independent, requires holiday junction resolvases, and may involve G-quadruplex DNA [42]–[44]. Our preliminary studies suggest that SpoVG may interact with other, as-yet unidentified factors. We are continuing studies to identify other players in the *vlsE* variation system in order to define the complicated mechanism of borrelial *vlsE* recombination. It is also possible that SpoVG*_Bb_* controls gene expression in *B. burgdorferi* as do the *S. aureus* and *B. subtilis* orthologs.

In conclusion, our data suggest that all SpoVG orthologues are DNA binding proteins. The two SpoVG homologs characterized in these studies, those of the firmicute *S. aureus* and the spirochete *B. burgdorferi*, each bound with high affinities to distinct DNA sequences. Those data suggest that, despite the overall similarities between SpoVG homologs of different species, each may preferentially bind to a different DNA sequence. The amino acid sequence of the SpoVG α-helix was found to be critical for DNA sequence-specificity. Two addition, invariant residues are essential for DNA-binding, probably through contacts with the negatively-charged DNA backbone. These results provide a framework upon which to define the roles of the ubiquitous SpoVG proteins in bacterial pathogenesis and cellular physiology.

## Materials and Methods

### Bacterial Strains

*S. aureus* strain Newman was cultured at 37°C in Luria Bertani (LB) broth with agitation. *B. burgdorferi* strain B31 was propagated in Barbour-Stoenner-Kelly (BSK)-II broth at 34°C [45]. Whole genomic DNAs from *B. burgdorferi* and *S. aureus* were purified using Qiagen genomic DNA extraction kits, following the manufacturer’s recommend procedure (Valencia, CA). Purified *L. monocytogenes* strain EGD-e genomic DNA was a gift from Dr. Sarah D’Orazio.

### DNA-affinity Chromatography

A protein was purified from *B. burgdorferi* cytoplasmic extract based on its affinity for *vlsE* DNA bait, using previously-described procedures [25], [27]. Bait DNA was generated by PCR of the *B. burgdorferi vlsE* coding region using one 5′-biotin-modified and one unmodified oligonucleotide (Table 2). A single band that eluted in buffer containing 750 mM NaCl was excised and MALDI-TOF MS/MS analysis was performed at the Clinical Proteomics Center, University of Louisville. Peptide masses were compared to the *B. burgdorferi* strain B31-MI sequence [46], [47], using Mascot (Matrix Science, Boston, MA). Significance parameters were fixed at p<0.05, corresponding to Ion score of 81.

**Table 2:** Oligonucleotides used in this study.

Name	Sequence (5′ 3′)	Target	Modification
Bio CapUp F-1	CTA TCT GAT AAT AAT CAT CTA ACT CAC	*capA5* 5′UTR	5′ Biotin
CapUp R-2	TAT TTA CCT CCC TTA AAA ATT TTC	*capA5* 5′UTR	None
CapUp F-1	CTA TCT GAT AAT AAT CAT CTA ACT CAC	*capA5* 5′UTR	None
fmtUp F-1	GTC ATC CTC CTG GTT GAT TAT TC	*fmtB* 5′UTR	None
fmtUp R-2		*fmtB* 5′UTR	None
lukEUp F-1	CTT AAA CAT AAG TTT CAC TTT CTT TC	*lukE* 5′UTR	None
lukEUp R-2		*lukE* 5′UTR	None
esxAUp F-1	CCT TTA TGT ATT TAA TTT AAT TTT AG	*esx* 5′UTR	None
esxAUp R-2	CCT CCT GAA TAT TTT AAG TTT ATC	*esx* 5′UTR	None
esxAIn F-1	CAC CAA TCC AGT TCA TTT CTG	*esx* ORF	None
esxAIn R-2	CCA AAG ATG GAC ACA ACG ATT AG	*esx* ORF	None
CapIn F-1	GCG CTA TTG TTA CAT TTT TCG TC	*capA5* ORF	None
CapIn R-2	GGT GAA TAC TTA TCA TTT AAG TCC	*capA5* ORF	None
BioSpo41	GAG TAT AAT TAT TTT TAA TTT ACA TAT AAA TAA AAA GGC GAA AAT AAT GCG GTT TAA AAG TAA TTA AT	*capA5* 5′UTR	5′ Biotin
Spo41-F	GAG TAT AAT TAT TTT TAA TTT ACA TAT AAA TAA AAA GGC GAA AAT AAT GCG GTT TAA AAG TAA TTA AT	*capA5* 5′UTR	None
Spo42-R	ATT AAT TAC TTT TAA ACC GCA TTA TTT TCG CCT TTT TAT TTA TAT GTA AAT TAA AAA TAA TTA ATA TAC TC	*capA5* 5′UTR	None
SpoMut33-F	GAG TAG CCG CCT TTT CGC CGG ACA TAT A	*capA5* 5′UTR	None
SpoMut34-R	TAT ATG TCC GGC GAA AAG GCG GCT ACT C	*capA5* 5′UTR	None
Bvls27F	GTT AAT AGT TTG CCT AAG G	*vlsE* ORF	5′ Biotin
Bvls77F	GTA CAG GTT CTG TTG GA	*vlsE* ORF	5′ Biotin
vlsR-10	ACC AAC AGA ACC TGT AC	*vlsE* ORF	None
vlsR-4	CTG GTT TCC CCG GTC GTA GTA C	*vlsE* ORF	None

### Recombinant Proteins

Purified *B. burgdorferi* B31 DNA was used as template to clone the borrelial *spoVG* gene into pET101, creating pBLJ132. Similarly, the *S. aureus* and *L. monocytogenes spoVG* genes were individually cloned into pET101 (Invitrogen, Grand Island, NY), producing pBLJ505 and pBLJ340, respectively. Each cloned insert was completely sequenced to confirm that the *spoVG* gene was free of mutations and in-frame with the hexa-histidine tag. *Escherichia coli* Rosetta-2 (Novagen, EMB Millipore, Billerica, MA) was independently transformed with pBLJ132, pBLJ340, or pBLJ505. Recombinant proteins were induced by the addition of 1 mM IPTG, and purified using MagnaHis Ni-Particles (Promega, Madison, WI). In order to create conditions conducive to protein-DNA interactions, each SpoVG protein was dialyzed against a buffer containing 100 nM dithiothreitol, 50 mM Tris-HCl, 25 mM KCl, 10% glycerol vol/vol, 0.01% Tween-20, 1 mM phenylmethanesulfonyl fluoride [25], [26], [29], [48]. Protein purities and concentrations were assessed via SDS-PAGE and Bradford analyses (Bio-Rad, Hercules, CA) respectively. Protein aliquots were snap frozen in liquid nitrogen and stored at −80°C.

To generate mutant SpoVG proteins, site-directed mutagenesis was performed on wild-type plasmid clones, as previously described [49]. Each plasmid was sequenced to confirm accuracy of mutations. All proteins were expressed, purified, and otherwise handled in the same manner. At least two independent protein preparations were used to evaluate each mutant protein that had a phenotypic difference from the wild-type protein. Tables 1 and 2 describe all probes, competitors, and mutant SpoVG proteins produced in this study follow the text.

### Electromobility Gel Shift Assays (EMSA)

Sequences of oligonucleotides used in this study are listed in Table 2. Oligonucleotide primers specific for the *B. burgdorferi vlsE* coding region or *S. aureus cap5* 5′ non-coding region were used to produce labeled probes, with one primer modified to include a 5′ biotin moiety that allowed for chemiluminescent detection. PCR-synthesized probes were purified by gel electrophoresis. Smaller, labeled DNA fragments were annealed by an initial high-temperature melting step, followed by incremental decreases in temperature using a thermocycler [48].

Unlabeled competitor DNAs were also generated via PCR or by annealing oligonucleotides. Larger competitors, consisting of *S. aureus capA, fmtB, esxA,* and *lukED* 5′ non-coding DNAs, were PCR amplified, and cloned into pCR2.1 (Invitrogen, Grand Island, NY, USA), generating pBLJ506, 507, 508, and 509, respectively. Each plasmid was sequenced to ensure that the clones were free of mutations. These constructs were then used as templates for PCR generation of specific competitors (Table 2). Amplicons were separated by agarose gel electrophoresis and purified using Wizard DNA Clean-up Systems (Promega, Madison, WI) before use as EMSA competitors.

All probe and competitor DNA concentrations were determined spectrophotometrically. When appropriate, competitor concentrations and oligonucleotide annealing efficiencies were also confirmed using relative ethidium bromide-stained band intensity following electrophoresis through native 20% polyacrylamide gels (Invitrogen, Grand Island, NY).

EMSA conditions were essentially the same as those described previously [25], [26], [29], [48]. Protein-DNA binding buffer consisted of 50 mM Tris-HCl (pH 7.5), 1 mM dithiothreitol, 150 nM EDTA, 50 ng/ml poly dI-dC, 2µl/ml phosphatase inhibitor (Sigma, St Louis, MO, USA), 8µl/ml protease inhibitor cocktail 2 (Sigma, St. Louis, MO). For reactions involving cell extracts *B. burgdorferi* B31 MI-16, cells were pelleted, resuspended in the above-described buffer, lysed, and cellular debris cleared by centrifugation. All EMSAs were performed at room temperature (approx. 20°C). Probe concentrations were varied as noted in the text. DNA and protein-bound DNA complexes were separated by electrophoresis through native a 10% polyacrylamide TBE gels (Invitrogen), transferred to a nylon membrane (Thermo Scientific, Waltham, MA), and UV cross linked (Stratagene UV Stratalinker 1800, La Jolla, CA). Nucleic acid probes were detected via chemiluminscence (Thermo Scientific) and visualized by autoradiography. Band densitometry was assessed using ImageJ (http://rsbweb.nih.gov/ij) and disassociation constants (K_D_) determined as previously described [50].

### Bioinformatic Analyses

Promoter motif and structural analyses were performed using MEME (Multiple Em for Motif Elicitation) (http://meme.sdsc.edu/meme/cgi-bin/meme.cgi). SpoVG amino acid sequences were retrieved from GenBank consortium (http://www.ncbi.nlm.nih.gov/sites). Amino acid sequences were Muscle aligned (http://www.ebi.ac.uk/Tools/msa/muscle/), with gap penalties set at 10, and a minimum of 1000 iterations. Images and analysis were generated using Geneious (http://www.geneious.com).

Species, strain, and GenBank accession numbers used for the analysis shown in Figure 4 were as follows: *Staphylococcus aureus* Newman, NP_645270.1; *Staphylococcus epidermidis* ATCC 12228, NP_765840.1; *Abiotrophia defectiva* ATCC 49176, ZP_04452046.1; *Bacillus anthracis* G9241, ZP_00240564.1; *Bacillus cereus* ATCC 14579, NP_829950.1; *Bacillus megaterium* WSH-002, YP_005497349.1; *Bacillus subtilis* 168, NP_387930.1; *Bdellovibrio bacteriovorus* HD100, NP_969591.1; *Borrelia burgdorferi* B31, NP_212919.1; *Borrelia hermsii* DAH, YP_001884203.1; *Caldicellulosiruptor saccharolyticus* DSM 8903, YP_001179173.1; *Clostridium botulinum* ATCC 3502, YP_001256027.1; *Clostridium perfringens* SM101, YP_699747.1; *Corallococcus coralloides* DSM 2259, YP_005368402.1; *Desulfarculus baarsii* DSM 2075, YP_003806692.1; *Elusimicrobium minutum* Pei191, YP_001875395.1; *Haloplasma contractile* SSD-17B, ZP_08554794.1; *Heliobacterium modesticaldum* Ice1, YP_001679836.1; *Ilyobacter polytropus* DSM 2926, YP_003968016.1; *Kyrpidia tusciae* DSM 2912, YP_003587990.1; *Leptotrichia goodfellowii* F0264, ZP_06012807.1; *Listeria monocytogenes* EGD-e, NP_463727.1; *Macrococcus caseolyticus* JCSC5402, YP_002561317.1; *Myxococcus xanthus* DK 1622, YP_633228.1; *Prevotella dentalis* DSM 3688, EHO58682.1; *Stigmatella aurantiaca* DW4/3-1, YP_003955432.1; *Spirochaeta thermophile* DSM 6192, YP_003874257.1; and *Treponema denticola* ATCC 35405, NP_971945.1.

### Multimerization State of SpoVG

15% SDS PAGE and 10% native PAGE were used to evaluate denatured and native masses of SpoVG preparations. Following electrophoretic separation polyacrylamide gels were stained with Coomassie Brilliant Blue.

Size-exclusion column chromatography was also employed to assess SpoVG multimerization in solution. A Superdex 200 10/300 column (GE Healthcare, Catalog No. 17-5175-01) was prepared per the manufacturer’s instructions with a mobile phase consisting of 300 mM NaCl, 25 mM Na2HPO4 (pH adjusted to 7.0 with 5.0 M HCl), 1 mM NaN3, and 1% glycerol. The mobile phase was sterile filtered to 0.22 µm. The flow rate was set to 0.10 ml/minute and elution was monitored at A280. The elution of proteins was calibrated using standards of known molecular weight from GE Healthcare LMW and HML Gel Filtration Calibration Kits. (Catalog Nos. 28-4038-41 and 28-4038-42). First, the void volume (V_0_) of the column was determined by injection of 100 µl of 1 mg/ml blue dextran 2000 (2,000 kDa) in elution buffer with 5% glycerol. Protein standards consisting of thyroglobulin (669 kDa), ferritin (440 kDa), aldolase (158 kDa), conalbumin (75 kDa), ovalbumin (43 kDa), carbonic anhydrase (29 kDa), ribonuclease A (13.7 kDa), and bovine lung aprotonin (6.5 kDa) were individually prepared in elution buffer with 5% glycerol at 10 mg/ml. These standards were then diluted such that each individual protein had a concentration of 2.0 mg/ml and injected in 100 µl aliquots. The log of the molecular masses of these standards was then graphed against resulting elution volumes (V_E_) as V_E_/V_0_ to produce a linear calibration. Individual experimental protein samples were then run and compared to this calibration curve to estimate molecular mass. Two independent protein preparations were used for each analysis.
